# Microglia Are Dispensable for Developmental Dendrite Pruning of Mitral Cells in Mice

**DOI:** 10.1523/ENEURO.0323-23.2023

**Published:** 2023-11-08

**Authors:** Tetsushi Niiyama, Satoshi Fujimoto, Takeshi Imai

**Affiliations:** Graduate School of Medical Sciences, Kyushu University, 812-8582, Fukuoka, Japan

**Keywords:** barrel, dendrite pruning, microglia, mitral cells, olfactory system

## Abstract

During early development, neurons in the brain often form excess synaptic connections. Later, they strengthen some connections while eliminating others to build functional neuronal circuits. In the olfactory bulb, a mitral cell initially extends multiple dendrites to multiple glomeruli but eventually forms a single primary dendrite through the activity-dependent dendrite pruning process. Recent studies have reported that microglia facilitate synapse pruning during the circuit remodeling in some systems. It has remained unclear whether microglia are involved in the activity-dependent dendrite pruning in the developing brains. Here, we examined whether microglia are required for the developmental dendrite pruning of mitral cells in mice. To deplete microglia in the fetal brain, we treated mice with a colony-stimulating factor 1 receptor (CSF1R) inhibitor, PLX5622, from pregnancy. Microglia were reduced by >90% in mice treated with PLX5622. However, dendrite pruning of mitral cells was not significantly affected. Moreover, we found no significant differences in the number, density, and size of excitatory synapses formed in mitral cell dendrites. We also found no evidence for the role of microglia in the activity-dependent dendrite remodeling of layer 4 (L4) neurons in the barrel cortex. In contrast, the density of excitatory synapses (dendritic spines) in granule cells in the olfactory bulb was significantly increased in mice treated with PLX5622 at postnatal day (P) 6, suggesting a role for the regulation of dendritic spines. Our results indicate that microglia do not play a critical role in activity-dependent dendrite pruning at the neurite level during early postnatal development in mice.

## Significance Statement

Synapse elimination is essential for activity-dependent circuit remodeling in the developing brains of mammals. Recent studies suggested that microglia play a critical role in the synapse elimination in some systems. This study found that microglia are dispensable for the activity-dependent dendrite pruning in developing mitral cells and layer 4 (L4) neurons in the barrel cortex. Thus, microglia are not critical for activity-dependent dendrite pruning at the neurite level during normal developmental process.

## Introduction

During the development of the mammalian nervous system, neurons initially form excessive synaptic connections. During early postnatal development, however, neurons undergo activity-dependent circuit remodeling to form functional neuronal circuits: they strengthen some neurites and synapses while eliminating others (Goodman and Shatz, 1993; Katz and Shatz, 1996). Activity-dependent circuit remodeling is also happening during the learning process in the adults, but at finer-scales (e.g., remodeling of dendritic spines). Elimination of neurites and synapses is critical to establish precise connectivity; however, molecular and cellular mechanisms of the elimination are poorly understood (Wong and Ghosh, 2002; Luo and O’Leary, 2005; Riccomagno and Kolodkin, 2015).

During early development, circuit remodeling often occurs at the neurite level. Both axons and dendrites are pruned during the remodeling process. In the neuromuscular junctions and climbing fiber, Purkinje cell synapses, for example, multiple axons connect to a single postsynaptic site, but eventually, all but only one axon is pruned (Lichtman and Colman, 2000; Watanabe and Kano, 2011). Similarly, over 20 retinal ganglion neurons connect their axons to a single neuron in the lateral geniculate nucleus in the early postnatal period; however, eventually, all but one to three strong connections are eliminated within two weeks after eye opening (Chen and Regehr, 2000).

In other systems, dendrites are dynamically pruned. Mitral cells in the olfactory bulb initially extend multiple dendrites to multiple glomeruli; however, they eliminate all but one primary dendrite, which is connected to only one glomerulus (Malun and Brunjes, 1996; Lin et al., 2000; Imai, 2014; Aihara et al., 2021; Fujimoto et al., 2023). This process ensures that each mitral cell receive inputs from a single type of olfactory sensory neurons (OSNs) expressing a specific type of odorant receptor. Layer 4 (L4) neurons in the barrel cortex are also known to establish single barrel-specific connectivity through the activity-dependent remodeling process (Erzurumlu and Gaspar, 2012; Nakazawa and Iwasato, 2021). L4 neurons eventually receive inputs from a single whisker. To form a discrete receptive field, both mitral cells and L4 neurons undergo dendrite pruning. In both neurons, NMDA receptor (NMDAR)-dependent synaptic competition mediates the dendrite pruning process. The failure in the dendrite pruning leads to broader receptive fields.

Recent studies indicate that glia play important roles in the activity-dependent synapse elimination. They exhibit phagocytotic activity and mediate synapse elimination during development (Brown and Neher, 2014; Thion et al., 2018; Faust et al., 2021). During the remodeling of retinal ganglion cell axons, microglia are reported to engulf their synapses in an activity-dependent manner (Stevens et al., 2007; Schafer et al., 2012). It is also known that the number of dendritic spines is increased by the genetic or pharmacological depletion of microglia, suggesting its requirement for the spine elimination (Paolicelli et al., 2011). Therefore, it is an attractive possibility that microglia mediate developmental dendrite pruning via its phagocytotic activity. However, it remains unclear whether microglia play a critical role in dendrite pruning during the developmental process.

In this study, we investigated the possible role of microglia in developmental dendrite pruning in mitral cells in mice. To selectively deplete microglia, we administrated a colony-stimulating factor 1 receptor (CSF1R) inhibitor, PLX5622. However, we did not find any apparent defects in the developmental dendrite pruning of mitral cells in mice treated with PLX5622.

## Materials and Methods

### Animals

All animal experiments were approved by the Institutional Animal Care and Use Committee of Kyushu University. Pregnant ICR mice were purchased from Japan SLC. Mice were kept under a consistent 12/12 h light/dark cycle (lights on at 8 A.M. and off at 8 P.M.). Both males and females were used for our experiments.

### Drug administration

Two types of CSF1R inhibitors, PLX3397 (MedChemExpress, #HY-16749) and PLX5622 (MedChemExpress, #HY-114153), were tested to deplete microglia. We injected the inhibitors into pregnant mice intraperitoneally to deliver them to embryos. After birth, inhibitors were injected intraperitoneally into the neonates. Based on pilot experiments (Extended Data Fig. 1-1*B*), PLX3397 was intraperitoneally injected at a dose of 20 mg/kg body weight twice a day for consecutive days to pregnant mothers (before birth) or pups (after birth). In accordance with previous studies (Rosin et al., 2018), PLX5622 was intraperitoneally injected at a dose of 50 mg/kg body weight once a day for consecutive days to pregnant mothers (before birth) or pups (after birth). In our pilot experiments, PLX3397 treatment reduced microglia at ∼70%, which was much lower than PLX5622. We, therefore, only analyzed data with PLX5622 treatment.

### Plasmids

pCAG-Flpo (3–10 ng/μl; Addgene, #125576), pCAFNF-tdTomato (1 μg/μl; Addgene, #125575), and pCAFNF-PSDΔ1.2-EGFP (1 μg/μl; Addgene, #125581) used for the *in utero* electroporation have been reported previously (Fujimoto et al., 2023).

### *In utero* electroporation

*In utero* electroporation was performed to label mitral cells at E12, granule cells at E13, and cortical L4 neurons at E13.5 as described previously (Matsui et al., 2013; Mizuno et al., 2014; Figueres-Oñate and López-Mascaraque, 2016; Fujimoto et al., 2023). Pregnant mice were anesthetized with ketamine (64–80 mg/kg) and xylazine (11–14 mg/kg). A total of 2 μl of plasmid solutions were injected into the lateral ventricle and electric pulses (a single 10-ms poration pulse at 72 V, followed by five 50-ms driving pulses at 32 V with 950-ms intervals) were delivered with forceps-type electrodes (3 mm in diameter, LF650P3, BEX) and a CUY21EX electroporator (BEX).

### Preparation of mouse brain samples

Mice were deeply anesthetized by intraperitoneal injection of pentobarbital (Sankyo, #P0776) and perfused with 4% paraformaldehyde (PFA) in PBS. The brain samples were fixed with 4% PFA/PBS overnight at 4°C. We embedded brain samples in 4% agarose in PBS and prepared brain slices (520 μm thick). To prepare cryosections, we incubated brain samples overnight in 20% sucrose and then in 30% sucrose with a gentle shake and embedded the samples in O.C.T. compound. Coronal frozen sections (16 μm thick) were prepared with a cryostat (Leica #3050S).

### Immunohistochemistry

Frozen sections were blocked with 5% skim milk and 1% Triton X-100 in PBS for 1 h at room temperature. Then, the sections were incubated with primary antibodies overnight at 4°C. Rabbit anti-Iba1 (1:1000, Wako, #019-19741), goat anti-OMP (1:500, Wako, #019-22291), rabbit anti-Homer1 (1:200, Synaptic Systems, #160003) were used as primary antibodies. After washing in PBS, sections were incubated with Alexa 555 or Alexa 488-conjugated donkey anti-rabbit or goat IgG (1:1000 for anti-Iba1 and 1:250 for others; ThermoFisher, #A31572, #A21432, and #A21206) for 2 h at room temperature. Nuclei were stained with DAPI (DOJINDO, #D523). To label a subset of olfactory sensory neurons, biotin-conjugated Lectin from Dolichos biflorus (1:1000, Sigma, #L6533-5MG) was used. Alexa Fluor 488-conjugated streptavidin (ThermoFisher, #S32354) was used to visualize the lectin signals.

For the analysis of the barrel cortex, 120-μm-thick tangential vibratome sections were prepared. The brain slices were incubated in blocking solution (2% saponin, 0.25% fish gelatin, 0.5% skim milk, 0.5% Triton X-100 and 0.05% sodium azide in PBS) overnight. Slices were then incubated with Guinea pig anti-VGluT2 (1:500, Millipore, AB2251-I) diluted in the blocking solution for 2 d at room temperature. After three times washing in 0.1% Triton X-100/PBS for 1.5 h, the slices were incubated with Alexa Fluor 488-conjugated donkey anti-guinea pig IgG (1:500, ThermoFisher, A-11073) diluted in the blocking solution for 2 d. After twice washing with 0.1% Triton X-100/PBS for 2 h, the slices were cleared with SeeDB2G.

### Tissue clearing with SeeDB2G

Fixed brain slices were stained with 0.1% DAPI in PBS overnight at room temperature. The brain samples were then cleared with SeeDB2G as described previously (Ke et al., 2016; Ke and Imai, 2018). Cleared samples were mounted with SeeDB2G on a glass slide with a 500-μm-thick rubber sheet and a coverslip.

### Confocal imaging and image processing

**Figure 1.**
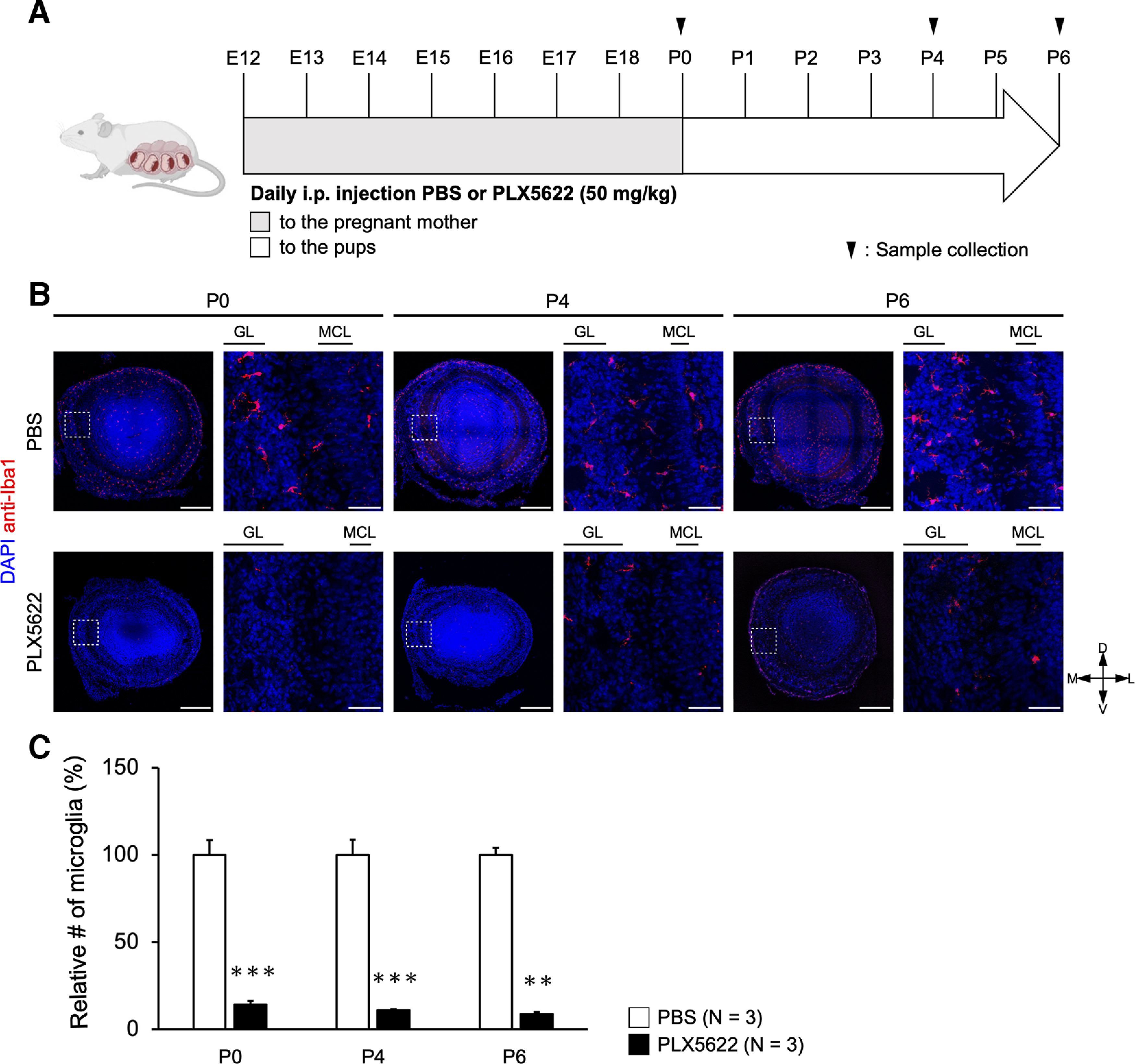
Depletion of microglia with PLX5622. ***A***, Timeline of PLX5622 administration. PLX5622 was intraperitoneally injected into pregnant mothers once a day from E12 and to the pups once a day from P0. Animals were analyzed at P0, P4, and P6 based on previous studies (Fujimoto et al., 2023). ***B***, Distribution of microglia in the olfactory bulb of control (top) and PLX5622-treated (bottom) mice. Anti-Iba1 staining shows microglia. GL, glomerular layer; MCL, mitral cell layer. Scale bars are 300 and 50 μm. D, dorsal; V, ventral; M, medial; L, lateral. ***C***, Quantification of Iba1-positive microglia in the olfactory bulb. One olfactory bulb per animal was analyzed. Every three sections (i.e., every 48 μm) were analyzed for each olfactory bulb for quantification (28–56 sections of 16-μm thickness per animal). Data are from three mice each. ***p <* *0.001, **p <* *0.01 (Welch’s *t* test). See also Extended Data Figures 1-1 and 1-2 for pilot experiments with PLX3397.

**Figure 2.**
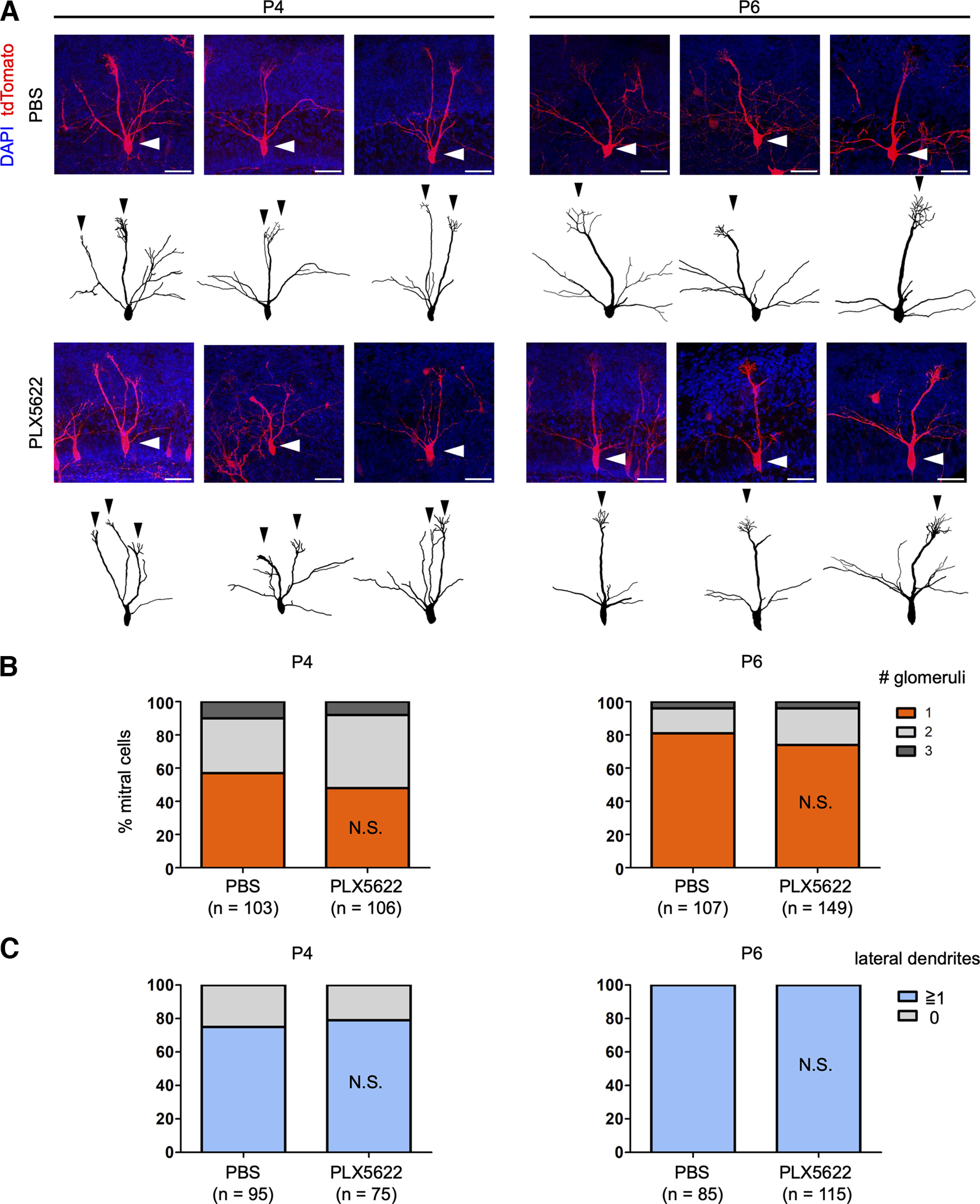
Microglial depletion does not affect dendritic pruning of mitral cells. ***A***, Representative images and reconstructions of mitral cells in control and PLX5622-treated mice at P4 and P6. White and black arrowheads indicate somata and primary dendrites of mitral cells, respectively. Scale bars represent 50 μm. ***B***, Quantification of the number of glomeruli innervated by single mitral cells. ***C***, Quantification of lateral dendrite formation. In this quantification, we excluded mitral cells whose lateral dendrites were densely labeled and difficult to trace. N.S., nonsignificant (χ^2^ test compared with the control). Number of neurons (*n*) are indicated in parentheses. Data are from five or six mice per group.

**Figure 3.**
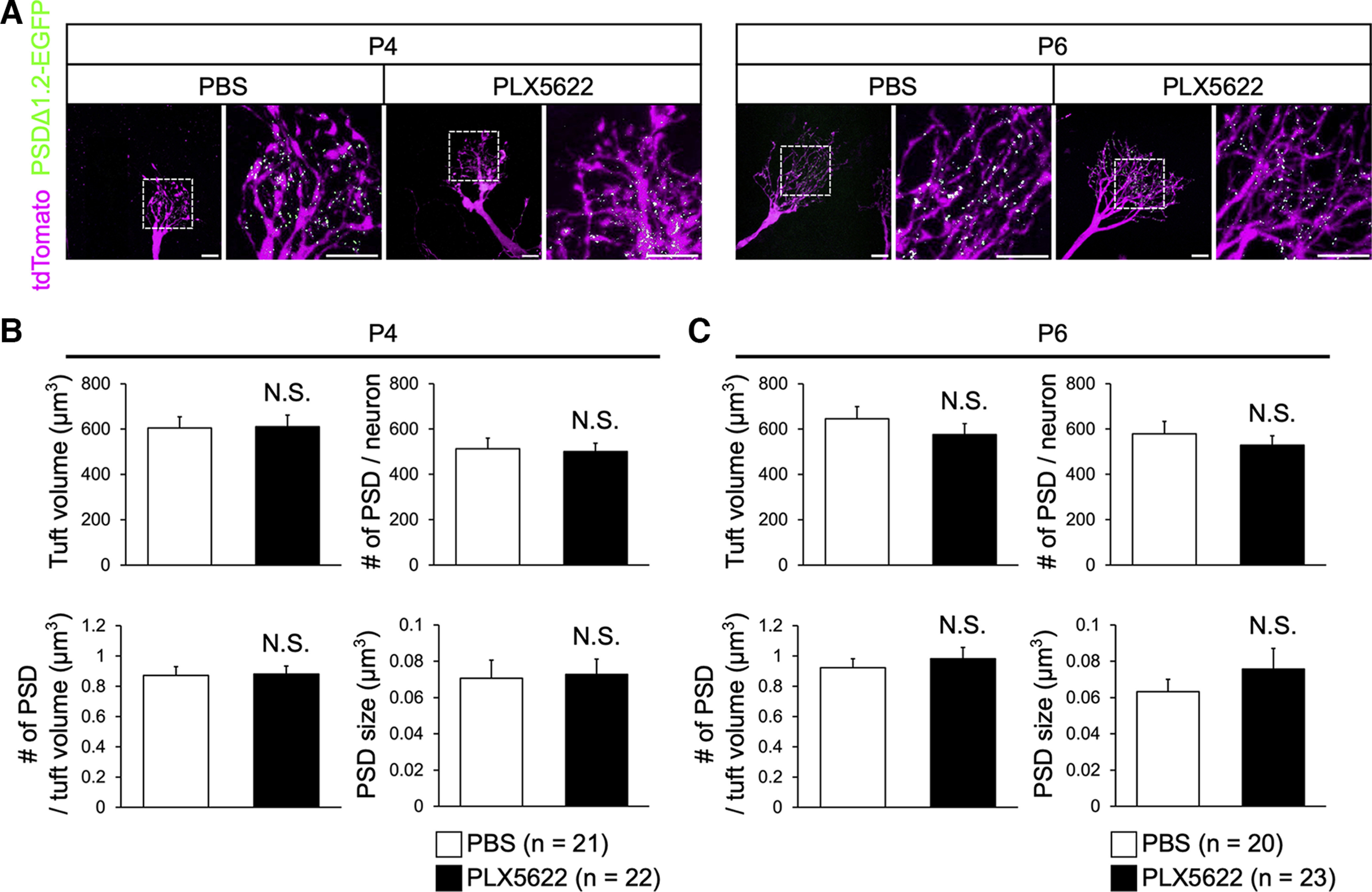
Microglial depletion does not affect the formation of excitatory synapses in mitral cells. ***A***, Representative images of dendritic tufts and PSDΔ1.2-EGFP puncta (excitatory synapses) of mitral cells in the control and PLX5622-treated mice at P4 and P6. Images are from glomerular layer. Scale bars represent 10 μm. ***B***, ***C***, Quantification of dendritic tuft volumes, number of PSD puncta, PSD puncta density, and PSD size at P4 (***B***) and P6 (***C***). N.S., nonsignificant (Welch’s *t* test). Number of neurons (*n*) are indicated in parentheses. Data are from five or six mice per group.

**Figure 4.**
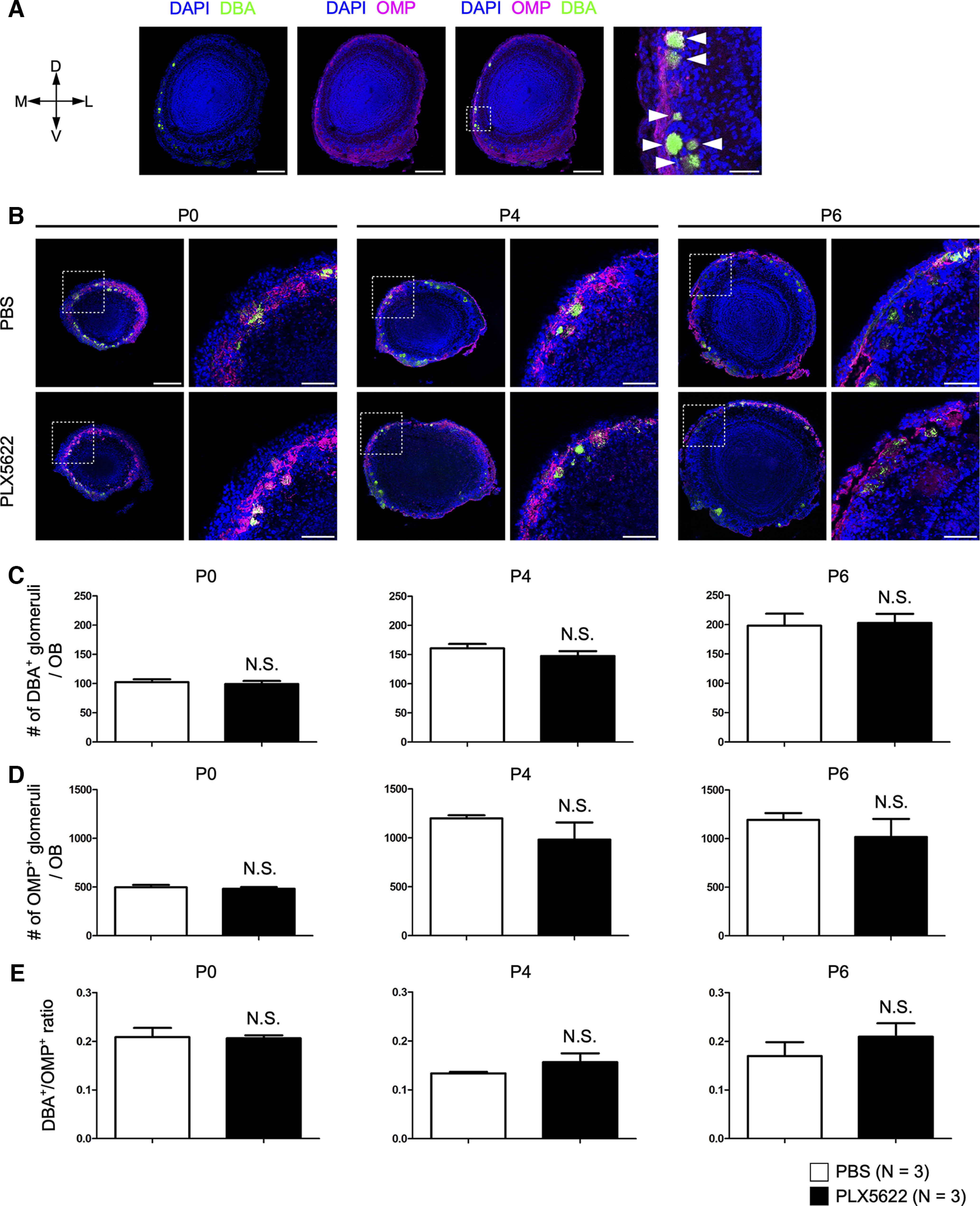
Microglial depletion does not affect axonal projection of OSNs. ***A***, Labeling a subset of OSN axons with DBA lectin. Numbers of OMP-positive and DBA-positive (white arrows) glomeruli were quantified. Scale bars are 300 μm (left) and 50 μm (right). D, dorsal; V, ventral; M, medial; L, lateral. ***B***, OSN projection in control (top) and PLX5622-treated (bottom) mice. Scale bars are 300 μm (left) and 100 μm (right). ***C–E***, Numbers of DBA-positive glomeruli (***C***), OMP-positive glomeruli (***D***), and DBA/OMP ratio (***E***). One olfactory bulb (hemibrain) per animal was analyzed for quantification every three sections (i.e., sampled every 48 μm). N.S., nonsignificant (Welch’s *t* test). Number of animals (*N*) are shown in parentheses.

**Figure 5.**
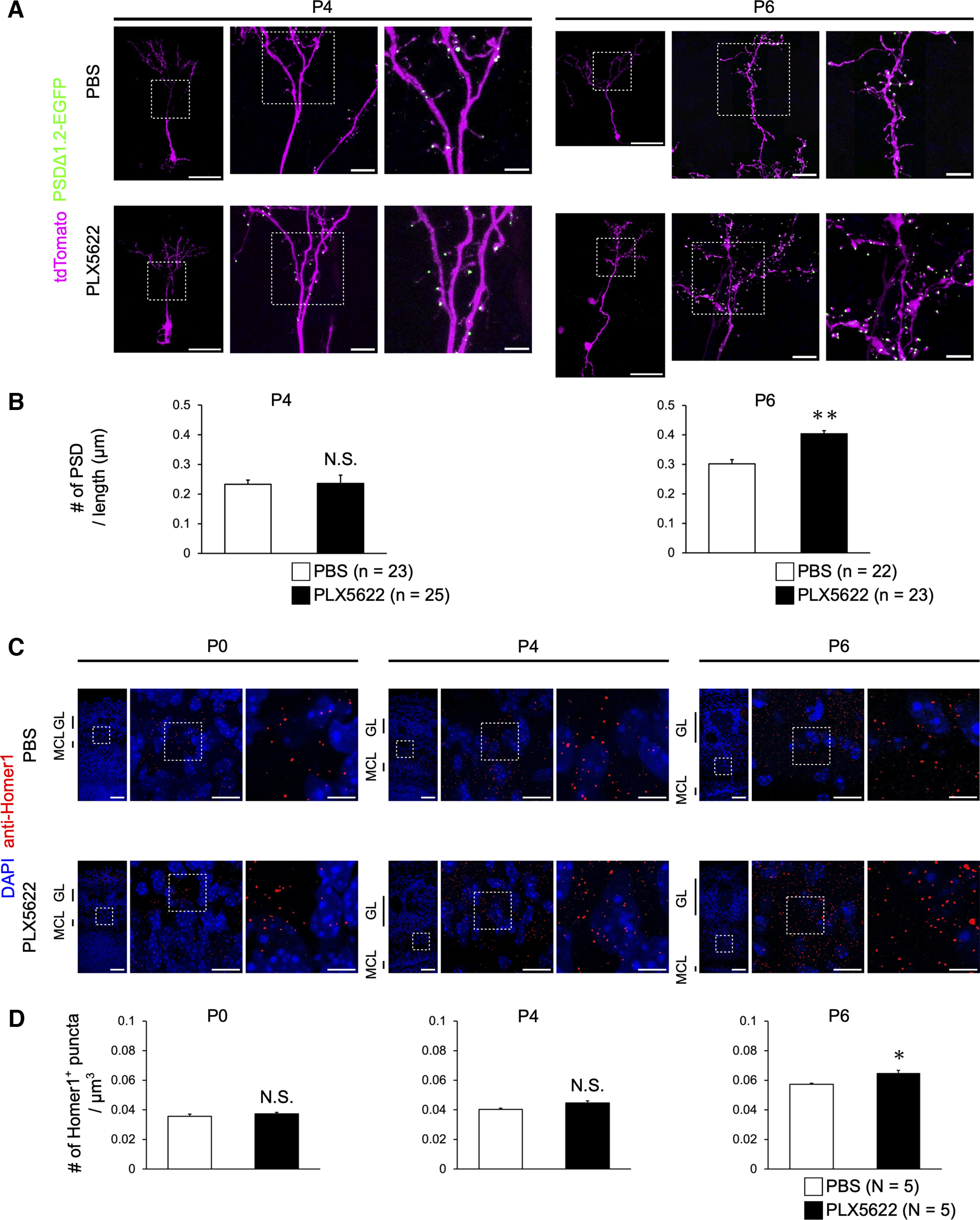
Microglial depletion increases the number of excitatory synapses in the olfactory bulb granule cells. ***A***, PSDΔ1.2-EGFP puncta in the granule cells in the olfactory bulb of control and PLX5622-treated mice. PSDΔ1.2-EGFP was introduced to a subset of granule cells using *in utero* electroporation at E13. Scale bars represent 50 μm (left), 10 μm (middle), and 5 μm (right). ***B***, Quantification of PSDΔ1.2-EGFP puncta in granule cells. **p <* *0.01, N.S., nonsignificant (Welch’s *t* test). Number of neurons (*n*) are shown in parentheses. Data are from three or four mice per group. ***C***, Excitatory synapses were visualized with anti-Homer1 antibody. Olfactory bulb sections of control (top) and PLX5622-treated (bottom) mice are shown. Scale bars represent 50 μm (left), 15 μm (right top), and 5 μm (right bottom). GL, glomerular layer; MCL, mitral cell layer. ***D***, Quantification of Homer1^+^ puncta density in the external plexiform layer of the olfactory bulb per volume. The density of Homer1^+^ puncta was unchanged at P0 and P4 but was significantly increased at P6. *p <* *0.05 (Welch’s *t* test). Number of animals (*N*) are shown in parentheses.

**Figure 6.**
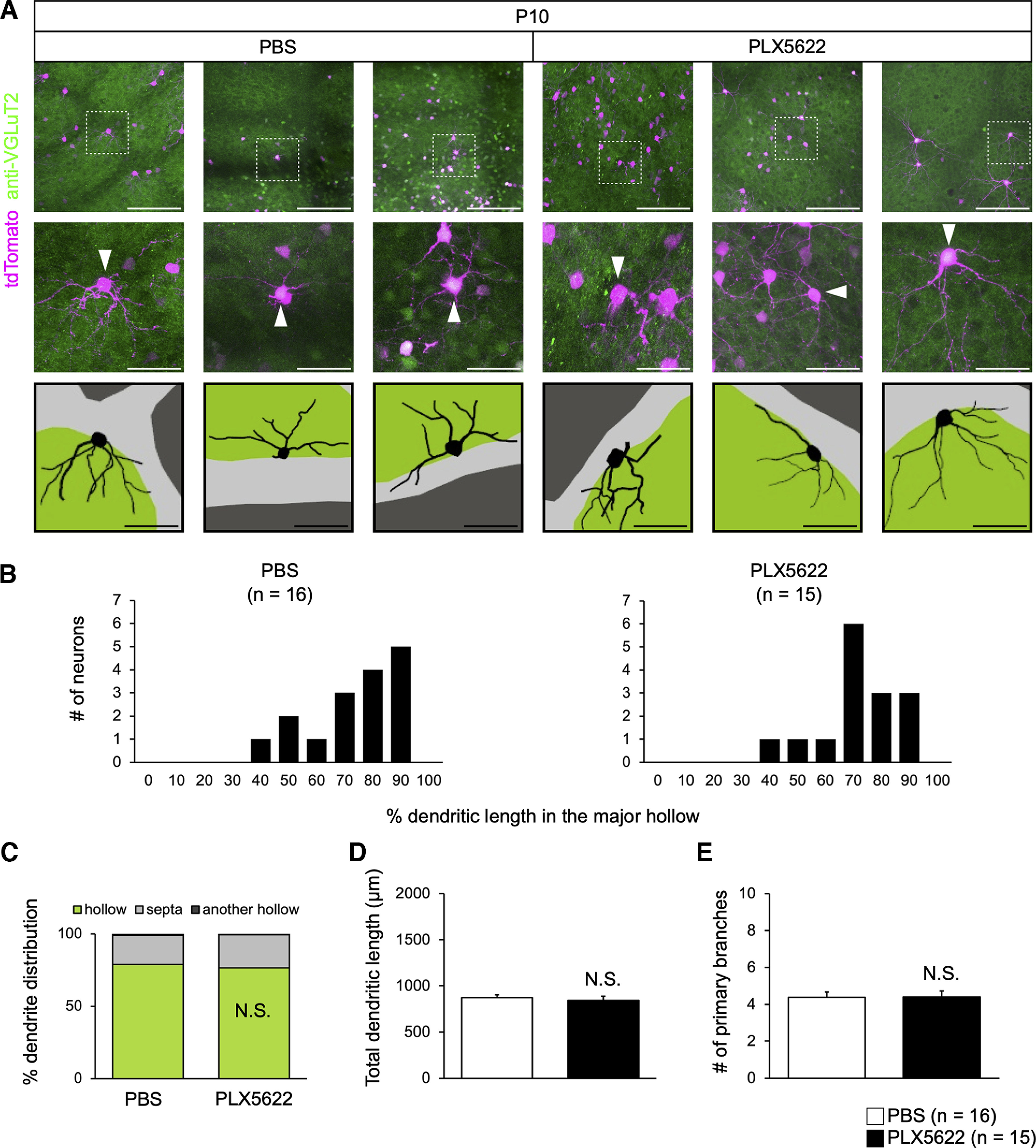
Microglial depletion does not affect the dendrite pruning of L4 neurons in the barrel cortex. ***A***, Representative dendritic reconstruction of L4 neurons in the barrel cortex. Barrel hollow (green or dark gray) was identified by anti-VGluT2 immunostaining. White arrowheads indicate somata of analyzed L4 neurons. Scale bars are 150 μm (top) and 50 μm (middle and bottom). ***B***, Dendritic orientation. The *x*-axis shows the percentage of the dendritic length found within the major follow. The *y*-axis shows the number of neurons. ***C***, Quantifications of dendritic orientation. Mean ratios (%) of dendritic length in the major follow (green), septa (light gray), and adjacent hollows (dark gray) are shown. N.S., nonsignificant (Welch’s *t* test). ***D***, Quantification of total dendritic length. N.S., nonsignificant (Welch’s *t* test). ***E***, Quantification of the number of primary branches. N.S., nonsignificant (Welch’s *t* test). Number of neurons (*n*) are shown in parentheses. Data are from three mice per group.

Fluorescence images were taken with a confocal microscope (Leica SP8) with a 20× (HC PL APO 20×/0.75 IMM CORR CS2, NA 0.75) or a 63× (HC PL APO 63×/1.3 Gly CORR CS2, NA 1.3) objective. Pixel size was 0.090 (Figs. 3, 5*C*), 0.284 (Figs. 1, 6; Extended Data Figs. 1-1*C*, 1-2*B*), and 0.568 μm/pixel (Figs. 2, 4, 5*A*; Extended Data Figs. 1-1*E*, 1-2*D*). Z-step size was 0.12 μm (Figs. 3, 5*A*,*C*), 1 μm (Figs. 1, 4, 6; Extended Data Figs. 1-1*C*, 1-2*B*), and 5 μm (Fig. 2; Extended Data Fig. 1-1*E*, 1-2*D*); a 405-nm laser was used for DAPI, a 488-nm laser was used for Alexa488 and GFP, and a 552-nm laser was used for Alexa555 and mRuby3.

10.1523/ENEURO.0323-23.2023.f1-1 Extended Data Figure 1-1 Microglial depletion by postnatal PLX3397 treatment. ***A***, Timeline of PLX3397 treatment in postnatal mice. PLX3397 was intraperitoneally injected to pups twice a day from P0 to P6. Mice were analyzed at P0, P4, and P6. ***B***, Relative number of microglia at P6 after treatment with different doses of PLX3397. The x-axis shows the dose of PLX3397 injection (mg/kg body weight). ***p <* *0.001, **p <* *0.01, *p <* *0.05 (Welch’s *t* test, compared with the control). ***C***, Anti-Iba1 staining in the olfactory bulb of control and PLX3397-treated (20 mg/kg body weight) mice. Scale bars represent 300 μm (left) and 50 μm (right). ***D***, Relative number of microglia at different stages. ***p <* *0.001, *p <* *0.05 (Welch’s *t* test, compared with the control). ***E***, Representative traces of mitral cells in control and PLX3397-treated mice. ***F***, Quantification of glomeruli innervated by individual mitral cells. N.S., nonsignificant (χ^2^ test compared to the control). Number of neurons (*n*) are indicated in parentheses. Data are from three or four mice per group. Download Figure 1-1, TIF file.

10.1523/ENEURO.0323-23.2023.f1-2 Extended Data Figure 1-2 Microglial depletion by PLX3397 treatment from embryonic stages. ***A***, Timeline of PLX3397 treatment from embryonic stages. PLX3397 was intraperitoneally injected into the pregnant mother twice a day from E6 and pups after birth. Mice were analyzed at P0, P4, and P6. ***B***, Anti-Iba1 staining in the olfactory bulb of control and PLX3397 (20 mg/kg body weight) treated mice. Scale bars represent 300 μm (left) and 50 μm (right). ***C***, Relative number of microglia at different stages. ***p <* *0.001, **p <* *0.01, *p <* *0.05 (Welch’s *t* test, compared with the control). ***D***, Representative traces of mitral cells in control and PLX3397-treated mice. ***E***, Quantification of glomeruli innervated by individual mitral cells. N.S., nonsignificant (χ^2^ test compared to the control). Number of neurons (*n*) are indicated in parentheses. Data are from three mice per group. Download Figure 1-2, TIF file.

### Quantification of neuronal morphology and synapses

Mitral cells located at the medial side of the olfactory bulb were analyzed for dendritic morphology following previous studies (Aihara et al., 2021; Fujimoto et al., 2023). Dendrites of mitral cells were reconstructed with Neurolucida (MBF Biosciences). The number of PSDΔ1.2-GFP or Homer1 puncta was counted using an ImageJ plugin, 3D Objects Counter (https://imagej.nih.gov/ij/plugins/track/objects.html).

### Statistical analysis

Prism7 (GraphPad) was used for statistical analysis. Data were acquired from at least three animals. Number of neurons (*n*) or animals (*N*) are indicated as a sample size within the figures. Number of animals used in the experiments are indicated in figure legends. Welch’s *t* test or χ^2^ test was used for statistical analyses.

### Data availability

Raw microscopy images have been deposited to SSBD:repository (https://doi.org/10.24631/ssbd.repos.2023.10.326).

## Results

### Depletion of microglia in the olfactory bulb by PLX5622 treatment

Microglial precursors are known to be generated in the yolk sac and infiltrate into the brain during the embryonic period, where they differentiate and produce microglia (Ginhoux et al., 2010). First, we examined the distribution of microglia in the olfactory bulb during the early postnatal stage with anti-Iba1 staining. We found that microglia are widely distributed in the olfactory bulb at postnatal day (P)0, before the dendrite pruning of mitral cells (Fig. 1*B*).

To investigate the role of microglia in the circuit remodeling in the olfactory bulb, we depleted microglia by drug administration. We used PLX5622, a selective and potent inhibitor of colony-stimulating factor 1 receptor (CSF1R), to remove microglia in the brain (Dagher et al., 2015). CSF1R is specifically expressed in microglia in the brain and is essential for microglial survival and proliferation. Therefore, inhibition of CSF1R can specifically deplete microglia (Stanley and Chitu, 2014). PLX5622 administration is known to deplete 80–90% of microglia after 3 d and over 90% after 7 d (Rosin et al., 2018). PLX5622 (50 mg/kg body weight) was intraperitoneally injected into pregnant mother mice from embryonic day (E)12 and intraperitoneally injected into the pups after birth (Fig. 1*A*). PLX5622 was administered once a day for consecutive days. At postnatal days 0, 4, and 6, the number of microglia was analyzed with anti-Iba1 immunostaining. The number of Iba1-positive microglia was reduced by >90% in PLX5622-treated mice at all stages.

We also tested PLX3397, a conventional CSF1R inhibitor (Elmore et al., 2014). We administered PLX3397 during postnatal stages (P0–P6; Extended Data Fig. 1-1) or from early pregnancy (E6–P6; Extended Data Fig. 1-2) at a concentration of 20 mg/kg body weight twice a day for consecutive days. Approximately 70% of microglia were depleted under this condition. Based on these results, PLX5622 was used in subsequent experiments.

### Microglial depletion does not prevent dendrite pruning of mitral cells

To examine the dendrite pruning of mitral cells, we employed *in utero* electroporation (Fujimoto et al., 2023). We performed *in utero* electroporation at E12 to label mitral cells. To facilitate neuronal reconstruction, we labeled mitral cells sparsely using a small amount of FLPo plasmid (3–10 ng/μl) and FLP-dependent tdTomato plasmid (1 μg/μl). It has been known that mitral cells initially extend multiple primary dendrites to multiple glomeruli, but between P4 and P6, a majority of mitral cells establish only one primary dendrite connecting to one glomerulus through the dendritic remodeling process (Fujimoto et al., 2023). In this study, olfactory bulb samples at P4 or P6 were analyzed. Samples were cleared with SeeDB2G and imaged with confocal microscopy. The number of glomeruli innervated by individual mitral cells was quantified after Neurolucida reconstruction.

At P4, 57.3% and 48.1% of mitral cells connected primary dendrites to a single glomerulus in control and PLX5622-treated mice, respectively (*p *=* *0.3466, χ^2^ test). At P6, 81.3% and 73.8% of mitral cells demonstrated single glomerular connection in control and PLX5622-treated mice, respectively (*p *=* *0.1255). We did not find statistical differences at both stages (Fig. 2). It should be noted that a small fraction of mitral cells retains multiple primary dendrites even at later stages (Fujimoto et al., 2023). Consistent results were obtained with PLX3397-treated animals (Extended Data Figs. 1-1, 1-2). It should also be noted that more dramatical pruning defects were found with smaller sample sizes by several genetic manipulations in our previous studies (e.g., NMDAR, RhoA, and BMPR2 knock-outs; Aihara et al., 2021; Fujimoto et al., 2023). Thus, microglia are dispensable for the developmental dendrite pruning in mitral cells.

### Microglial depletion does not affect the formation of excitatory synapses in mitral cell dendrites

As we did not find defects in dendrite pruning at the neurite level, we examined possible defects at finer scales. We compared the volume of dendritic tufts within the glomerulus, the number of postsynaptic density (PSD) per neuron, the number of PSD per dendritic tuft volume, and the size of PSD. We used a PSD marker, PSDΔ1.2-EGFP, introduced by *in utero* electroporation (Hayashi-Takagi et al., 2015; Fujimoto et al., 2023). As mitral cells do not have dendritic spines for excitatory synapses, PSDΔ1.2-EGFP is helpful in identifying functional excitatory synapses. However, none of them showed clear defects at both P4 and P6 in PLX5622-treated mice (Fig. 3). Thus, microglia are dispensable for the normal formation and pruning of dendritic tufts and PSDs in mitral cells during the early postnatal stages.

### Microglial depletion does not affect the axonal projection of OSNs

In the visual system, microglia are involved in presynaptic pruning. We, therefore, examined whether the projection and refinement of OSN axons are affected by microglial depletion. OSN axons form glutamatergic synapses onto the dendrites of mitral and tufted cells within the glomerulus. We used anti-OMP immunostaining to count the total number of glomeruli. We also examined the convergence and refinement of specific sets of OSNs using Lectin from Dolichos biflorus (DBA-lectin), which labels a subset of OSNs (St John and Key, 2002; Imai et al., 2009). In control animals, only 10–20% of glomeruli are innervated by DBA-positive OSN axons (Fig. 4*A*). We reasoned that the number of DBA-positive glomeruli should increase if the convergence of like axons is severely impaired. However, we did not find differences in the number of total as well as DBA-positive glomeruli by the PLX5622 treatment (Fig. 4*B*). There were no significant differences in the DBA/OMP ratio at all stages (*p *=* *0.9118 at P0, 0.2822 at P4, 0.3717 at P6, Welch’s *t* test; Fig. 4*C–E*). Thus, the axonal projection of OSNs is largely intact without microglia.

### Synaptic development of granule cells is affected by the microglial depletion

In the olfactory bulb, >90% of neurons are inhibitory interneurons, among which granule cells are the most abundant. These interneurons migrate through the rostral migratory stream and are continuously integrated into the olfactory bulb, even in the adult. Lateral dendrites of mitral cells form reciprocal synapses with granule cells in an activity-dependent manner (Kelsch et al., 2009; James et al., 2017). It has been previously reported that microglial depletion in adults leads to a reduction in spine density and elevation of spine head size in adult-born granule cells (Wallace et al., 2020).

We first examined the formation of excitatory synapses at the dendritic spines of granule cells using a PSD marker, PSDΔ1.2-EGFP. We sparsely introduced PSDΔ1.2-EGFP with a filler, tdTomato, using *in utero* electroporation at E13 (Figueres-Oñate and López-Mascaraque, 2016). In this experiment, we labeled a subset of embryonically generated granule cells. PSDΔ1.2-EGFP signals were mostly located at the head of the dendritic spines. At P4, we found no difference in the PSD density between the control and PLX5622-treated mice (*p *=* *0.9456, Welch’s *t* test). However, the PSD density was increased by PLX5622 treatment at P6 (*p *= 0.0019, Welch’s *t* test), suggesting that microglia have a suppressive role for excitatory synaptogenesis in granule cells at a later stage (Fig. 5*A*,*B*).

To further examine the synaptogenesis in granule cells, we examined the density of Homer1-positive PSD in the external plexiform layer, where granule cell dendrites form excitatory synapses. Again, the Homer1-positive PSD density was significantly increased only at P6 by microglial depletion (*p *=* *0.7250 at P0, 0.6878 at P4, and 0.0260 at P6), consistent with the PSDΔ1.2-EGFP labeling experiment (Fig. 5*C*,*D*).

### Microglial depletion does not affect dendrite pruning in cortical L4 neurons

Lastly, we examined whether microglia are generally dispensable for activity-dependent dendrite pruning. Cortical L4 neurons are a classical model of activity-dependent developmental dendrite pruning (Goodman and Shatz, 1993; Wong and Ghosh, 2002; Matsui et al., 2013). In sensory areas, L4 neurons receive sensory inputs from the thalamus. In mice, L4 neurons in the barrel cortex show clear orientation bias toward the barrel hollow, representing inputs from a specific whisker. Early in the development, dendritic growth is unbiased, but early in the postnatal development, biased orientation is formed through dendrite remodeling. The remodeling process involves the spontaneous activity and NMDA receptor-dependent synaptic competition (Mizuno et al., 2014, 2018; Antón-Bolaños et al., 2019; Fujimoto et al., 2023). Thus, L4 neurons in the barrel cortex are another valuable model to study the dendrite pruning and formation of the discrete receptive field.

We used *in utero* electroporation at E13.5 to sparsely label L4 neurons in the barrel cortex and analyzed their morphology at P10. In both control and PLX5622-treated animals, ∼80% of their dendrites were confined within a specific hollow, identified by VGluT2 immunostaining (Fig. 6*A–C*). Moreover, we found no difference in the total dendritic length and the number of primary branches in L4 neurons (Fig. 6*D*,*E*). Thus, microglia are dispensable for the normal dendritic patterning of cortical L4 neurons in the mouse barrel cortex.

## Discussion

### Roles of microglia in the development of the olfactory bulb circuits

Activity-dependent circuit remodeling is essential for the refinement of the nervous system (Goodman and Shatz, 1993; Katz and Shatz, 1996). It is well understood how neurite outgrowth and stabilization are controlled; however, the mechanisms of the neurite pruning process are still poorly understood. Specifically, the fundamental question is what instructs the pruning process (Lichtman and Colman, 2000; Riccomagno and Kolodkin, 2015). In this study, we examined whether microglia play a critical role in the circuit remodeling in the olfactory bulb.

In the olfactory bulb, developmental dendrite pruning in mitral cells is essential to ensure the one mitral cell – one glomerulus rule, whereby sensory inputs from a single type of odorant receptor are conveyed to each mitral cell in the glomerulus (Imai, 2014). We found that microglial depletion does not perturb the normal dendrite pruning process in mitral cells (Fig. 2). Moreover, we found no change in the formation of excitatory synapses in the dendritic tufts of mitral cells (Fig. 3). Thus, microglia are dispensable for the developmental elimination of neurites and synapses in mitral cells.

In the visual circuits and hippocampus, microglia mediate presynaptic pruning (Faust et al., 2021; Guedes et al., 2022). In this study, we did not find apparent changes in OSN projection by microglial depletion based on DBA and OMP staining (Fig. 4). It is possible, however, that genetic labeling of a specific type of OSNs may reveal finer changes in the axonal convergence of OSNs.

In this study, we observed evident changes by microglial depletion only in the granule cells in the olfactory bulb. At P6, but not P0 or P4, the density of excitatory synapses (dendritic spines) in the granule cells was increased by microglial depletion, suggesting that microglia have a suppressive role in the excitatory synapse development (Fig. 5). As the number of excitatory synapses continued to increase both in the control and PLX5622-treated mice, it remains unclear whether the increase is because of the reduced synapse elimination or enhanced synaptogenesis. Microglia are reported to facilitate synaptogenesis in some situations (Thion et al., 2018; Guedes et al., 2022); however, we did not find differences in the initial phase of synaptogenesis in granule cells. Our results are consistent with a recent study of adult-born granule cells in the olfactory bulb (Wallace et al., 2020).

It should be noted that astrocytes and oligodendrocyte precursors are also reported to mediate synapse pruning (Chung et al., 2013; Tasdemir-Yilmaz and Freeman, 2014; Auguste et al., 2022; Buchanan et al., 2022, 2023). Therefore, our study does not fully exclude the role of glia and phagocytosis in the dendrite pruning in mitral cells.

We only examined the developmental process in this study. It is possible, however, that microglia play a critical role in circuit remodeling in the adult (Miyamoto et al., 2016), ischemia and injury models (Rappert et al., 2004; Murai et al., 2016), or under neurodegenerative diseases (Guedes et al., 2022) where inflammation by microglia could be more prominent. These are interesting issues for future studies.

It is still debated to which extent glial cells instruct the pruning process (Kettenmann et al., 2011; Riccomagno and Kolodkin, 2015). Glial cells may only eat neurites and synapses which are already committed to the degeneration process.

### Mechanisms of developmental dendrite pruning

During development, neurite outgrowth and pruning are both essential to form mature neuronal circuits. It has been suggested that neurite pruning is mediated either by degeneration or retraction (Luo and O’Leary, 2005). Stereotyped large-scale axon pruning is often mediated by degeneration mechanisms (Awasaki and Ito, 2004; Vanderhaeghen and Cheng, 2010; Riccomagno and Kolodkin, 2015). Stereotyped dendrite pruning during *Drosophila* metamorphosis is also mediated by degeneration: before metamorphosis, the stem of the dendrites is thinned by endocytosis, and then the entire dendrite is degenerated by caspase and calpain (Kanamori et al., 2013, 2015). However, this mechanism seems to be unique to insects. Axon pruning in the activity-dependent remodeling process may also involve axon degeneration in mice (Faust et al., 2021; Nagappan-Chettiar et al., 2023). In these cases, the degenerating neurites present the “eat-me” signals, and microglia mediate the clearance of degenerated neurites (Stevens et al., 2007; Schafer et al., 2012; Sapar et al., 2018; Scott-Hewitt et al., 2020; Faust et al., 2021). Microglia is also known to mediate pruning of postsynaptic structures (e.g., dendritic spines; Paolicelli et al., 2011). Indeed, granule cells in the olfactory bulb appear to involve similar mechanisms for synapse elimination (Grier et al., 2016; Denizet et al., 2017; Kurematsu et al., 2022).

In contrast, activity-dependent dendrite pruning at the neurite level in mammals appears to involve a retraction process mediated by actin cytoskeletal regulation. *In vivo* imaging of L4 neurons in the barrel cortex demonstrated that the dendrite patterning results from “trials and errors” of dendritic growth and retraction (Mizuno et al., 2014). Moreover, recent studies showed that Rho-family small GTPases Rac1 and RhoA mediate the stabilization and pruning of mitral cell dendrites, respectively, suggesting that remodeling of the actin cytoskeleton is the key (Aihara et al., 2021; Fujimoto et al., 2023). Indeed, we did not observe the fragmented debris of mitral cell dendrites in the developing olfactory bulb, even in the PLX5622-treated mice (Fig. 2). Our current study indicates that microglia are dispensable for activity-dependent dendrite pruning at the neurite level at this stage. Thus, microglia mediate only a specific type of synaptic pruning.

It is known that the activity-dependent pruning of neurites and synapses is a result of synaptic competition. In synaptic competition, a postsynaptic cell decides which inputs to maintain and eliminates all the others as losers based on the interplay between neurites (Fujimoto et al., 2023). Once the loser postsynapses are determined, the retrograde signals may facilitate the degeneration of input axons (Nagappan-Chettiar et al., 2023). Thus, we assume that intracellular signals within the postsynaptic cell instruct neurite elimination, not only in postsynapses, but also at presynapses. Elucidating the core molecules for synaptic competition should reveal the origin of the pruning specificity in neural development.

## Synthesis

Reviewing Editor: Matthew Grubb, King’s College London Decisions are customarily a result of the Reviewing Editor and the peer reviewers coming together and discussing their recommendations until a consensus is reached. When revisions are invited, a fact-based synthesis statement explaining their decision and outlining what is needed to prepare a revision will be listed below. The following reviewer(s) agreed to reveal their identity: Charles Greer, Ana Dorrego-Rivas.

Synthesis:

Both Reviewers are positive about the manuscript, and make suggestions for improvement. Please note that after consultation together, we agreed that further data *are not required* to address Reviewer 1’s comments regarding GC inputs onto M/TCs, nor their comments regarding apical glomerular arborisations. However, we do think it is important that data on the pruning/development of M/TC lateral dendrites is added to the manuscript. Please also address all of each Reviewer’s comments in full.

Reviewer 1:

In this potentially interesting report the authors use a pharmacological strategy to deplete microglia by approximately 90% from developing embryos and then continue the depletion postnatally. Using in utero electroporation at E12 they label olfactory bulb mitral cells examined their apical dendritic arbors at multiple ages. Their data show that while the previously described trimming or loss of supernumerary apical dendrites is not affected by the decrease in microglia nor does the uni-glomerular targeting of the apical dendrites appear affected. Similarly, they show that the density of synapses, presumably from olfactory sensory neurons, onto the apical dendrites within the glomeruli was not affected as measured by PSD density and that at least grossly, the targeting of the sensory neuron axons to individual glomeruli was not changed. The only effect they report is an increase in PSD density on granule cells. To further they studies, they also report that cortical L4 neuron dendrite pruning is not affected by the absence of microglia. It is interesting to note at this time that microglia are implicated in neurogenesis in the subventricular zone which gives rise to the granule cells that populate the olfactory bulb. It might have been predicted that alterations in the number or distribution of granule cells would have been an early indicator of a potential impact of microglia depletion.

Before going further I should also note that the data reported here is strikingly similar to data heavily cited in the current submission and previously reported by what I presume was this group, including the data on L4 neurons in cortex, and appeared in Developmental Cell https://doi.org/10.1016/j.devcel.2023.05.004

The results are clearly in contrast with prior reports, from the visual system for example, where microglia have been repeatedly implicated in dendritic pruning as well as the refinement of synaptic connections. The authors do not explore why microglia do not appear to be involved but do note that prior studies (they report as “their” studies) showed alternations in mitral cell pruning in NMDAR, RhoA and BMPR2 knockout mice. However, they do not include in the current manuscript a cogent discussion of why microglia apparently had not effect in the L4 cortical neurons or the olfactory system while they seem to be a potent mediator of pruning elsewhere in the CNS. A discussion of this would be very helpful to many readers who may be less familiar with either CNS pruning or microglia.

Second, the results from the PSD increase in granule cells is provocative. The literature clearly established some time ago that single granule cell spines establish on 1 reciprocal dendrodendritic synapse. If there is an increase in the PSD associated with the labeled granule cells it seems unexpected that there would not be a compensatory increase in the inhibitory synapse from the granule cell back to the apposed mitral cell dendrite. Testing for changes in gephyrin labeling (the GABAR anchor) seems imperative in these studies.

Finally, I note that while pruning may not have affected the primary apical shaft of the mitral cell, the primary arbor is found within the glomerulus and these were not examined in the current study, which seems an oversight. Similarly, the secondar dendrites of the mitral cells which distribute horizontally and in some cases obliquely in the external plexiform layer are also subject to pruning during development but were not included in the analyses reported here.

Figures

I believe panel A in Fig. 1 may be in error. The PLX5622 was administered to the mother from E12 - PO but the shading of the legend says the opposite. In panel C, if the axis is showing % of microglia, how can there be more than 100% as indicated by the error bar at P6. Also, analyzing only 3 sections from each of 3 mice is a rather low sample rate.

The number of neurons reconstructed for Figure 2 looks fine, but how many mice were used? I could not find any clear discussion of how many mice were used in these studies. Three mice are reported for Figs. 1 and 4 while more are reported in Figure 5. No mention of whether both male and female mice were tested.

The PSD labeling in Figure 5 is very difficult to resolve, even after increasing the magnification by 400%.

Figure 6 - the addition of the data from the L4 cortical neurons does not add substantively to the paper since the timing of their development and the embryonic origin differs from that of the olfactory bulb neurons. Insight into the mechanisms involved is not clarified by the addition of the data from L4.

Reviewer 2:

Mitral cells in the olfactory bulb and neurons in the barrel cortex undergo dendritic remodelling during development. One potential candidate to regulate this process is microglia, as it has been showed to be involved in synaptic pruning. Here, the authors convincingly demonstrate that microglia are not involved in dendritic and synaptic pruning in mitral cells and dendritic pruning in neurons in the barrel cortex. However, they found that microglia does regulate synaptic remodelling in granule cells in the olfactory bulb.

The findings are novel, interesting and useful for the field, and are based on robust data. The authors thoroughly explored every possible scenario in terms of pruning in mitral cells (dendritic, presynaptic, postsynaptic), delved into the olfactory circuitry (granule cells) and investigated the same process in another neuron type, in this case the neurons of the barrel cortex. For all the reasons above, I strongly recommend this article for publication.

I have several minor comments:

- Please, replace any red channel in the figures by magenta, or another colour-blind friendly choice (like in Fig. 3).

- Fig. 1A, Supp. Fig. 1A, and Supp. Fig. 2A: please, specify ‘ICR’ abbreviation in figure legend.

- Supp. Fig. 1D, Supp. Fig. 2D: the authors should include the statistical tests in the quantifications. Were they not considered because they finally chose the most efficient inhibitor?

- Fig. 3: The authors show convincingly that dendritic pruning is unaffected between control and microglia-depleted conditions, both at P4 and P6. However, there are two percentages of neurons in the controls that do not end up projecting to a single glomerulus. Is this physiological/known? If so, could the authors indicate it briefly in the discussion/highlight the reference of the study?

- Fig. 2, Fig. 3, Fig. 6: for the sake of consistency with previous figures, please indicate the number of mice used in these experiments.

- Fig. 3: for the sake of clarity, please add the ages in graphs of B and C (like the ones in Fig. 4).

- Fig. 6: like in Fig. 2, please add example images of electroporated neurons to accompany the schematics of the reconstructions.

- Methods, ‘Confocal imaging and image processing’ section: details about type of acquisitions (e.g., stacks) and pixel size should be added.

## Author Response

Reviewer #1

In this potentially interesting report the authors use a pharmacological strategy to deplete microglia by approximately 90% from developing embryos and then continue the depletion postnatally. Using in utero electroporation at E12 they label olfactory bulb mitral cells examined their apical dendritic arbors at multiple ages. Their data show that while the previously described trimming or loss of supernumerary apical dendrites is not affected by the decrease in microglia nor does the uni-glomerular targeting of the apical dendrites appear affected. Similarly, they show that the density of synapses, presumably from olfactory sensory neurons, onto the apical dendrites within the glomeruli was not affected as measured by PSD density and that at least grossly, the targeting of the sensory neuron axons to individual glomeruli was not changed. The only effect they report is an increase in PSD density on granule cells. To further they studies, they also report that cortical L4 neuron dendrite pruning is not affected by the absence of microglia. It is interesting to note at this time that microglia are implicated in neurogenesis in the subventricular zone which gives rise to the granule cells that populate the olfactory bulb. It might have been predicted that alterations in the number or distribution of granule cells would have been an early indicator of a potential impact of microglia depletion.

Before going further I should also note that the data reported here is strikingly similar to data heavily cited in the current submission and previously reported by what I presume was this group, including the data on L4 neurons in cortex, and appeared in Developmental Cell https://doi.org/10.1016/j.devcel.2023.05.004

We thank this reviewer for careful reading of our manuscript.

The results are clearly in contrast with prior reports, from the visual system for example, where microglia have been repeatedly implicated in dendritic pruning as well as the refinement of synaptic connections. The authors do not explore why microglia do not appear to be involved but do note that prior studies (they report as “their” studies) showed alternations in mitral cell pruning in NMDAR, RhoA and BMPR2 knockout mice. However, they do not include in the current manuscript a cogent discussion of why microglia apparently had not effect in the L4 cortical neurons or the olfactory system while they seem to be a potent mediator of pruning elsewhere in the CNS. A discussion of this would be very helpful to many readers who may be less familiar with either CNS pruning or microglia.

We thank this reviewer for raising this point. Perhaps, the roles of microglia have often been overstated in previous literature. Therefore, we agree that it is important to include a consensus view in the Discussion section based on current and previous studies.

First, there are a number of studies showing that developmental “axon” pruning is mediated by microglia; however, there is no clear evidence showing that neurite-scale “dendrite” pruning is mediated by microglia during development. Second, microglia may mediate synaptic scale pruning of dendrites, i.e., dendritic spine pruning. However, activity-dependent dendrite pruning at the neurite level appears to be mediated by cytoskeletal regulation rather than phagocytosis by microglia. Finally, it is possible that microglia mediate neurite-scale pruning of dendrites only under neurological and/or inflammatory conditions, but not during normal development.

These points are now included in the Significance statement (Line 73-74) and Discussion (Line 408-424).

Second, the results from the PSD increase in granule cells is provocative. The literature clearly established some time ago that single granule cell spines establish on 1 reciprocal dendrodendritic synapse. If there is an increase in the PSD associated with the labeled granule cells it seems unexpected that there would not be a compensatory increase in the inhibitory synapse from the granule cell back to the apposed mitral cell dendrite. Testing for changes in gephyrin labeling (the GABAR anchor) seems imperative in these studies.

Granule cells form synapses not only with mitral/tufted cells, but also with centrifugal axons from the olfactory cortex. Thus, we would not be surprised if there was no compensatory increase in the inhibitory synapses in the lateral dendrites of mitral cells.

Furthermore, we focused on dendrite pruning in mitral cells in this study. We used granule cell data as a positive control experiment because effects on granule cells have already been documented by Wallace et al. (2020). Therefore, the suggested experiment, while appreciated, seems to be beyond the scope of this paper.

Finally, I note that while pruning may not have affected the primary apical shaft of the mitral cell, the primary arbor is found within the glomerulus and these were not examined in the current study, which seems an oversight. Similarly, the secondar dendrites of the mitral cells which distribute horizontally and in some cases obliquely in the external plexiform layer are also subject to pruning during development but were not included in the analyses reported here.

We have now included the quantitative data for lateral dendrites in Fig. 2C. Unfortunately, it was difficult to quantify the exact number of lateral dendrites in many samples, because the density of mitral cell labeling was not optimized for lateral dendrite quantification. In this analysis, we excluded mitral cells whose lateral dendrites were difficult to trace. As a result, sample size is smaller than in Fig. 2B.

Figures

I believe panel A in Fig. 1 may be in error. The PLX5622 was administered to the mother from E12 - PO but the shading of the legend says the opposite. In panel C, if the axis is showing % of microglia, how can there be more than 100% as indicated by the error bar at P6. Also, analyzing only 3 sections from each of 3 mice is a rather low sample rate.

We apologize for the error in Fig. 1A. This is now corrected.

In Fig. 1C, we quantified the total number of microglia in 1/3 of the olfactory bulb sections (28-56 sections per animal instead of 3). The “mean number” of microglia in the PBS control (N = 3 mice) is defined as 100%. Error bars represent variation within the 3 olfactory bulb samples. Similar quantification was performed in a previous study (Wallace et al., 2020).

For consistency of presentation, we have also updated the corresponding panels in Extended Data Figure 1-1D and 1-2C (see our response to Reviewer #2).

The number of neurons reconstructed for Figure 2 looks fine, but how many mice were used? I could not find any clear discussion of how many mice were used in these studies. Three mice are reported for Figs. 1 and 4 while more are reported in Figure 5. No mention of whether both male and female mice were tested.

The numbers of animals are now described in figure legends (typically 3-6). We used both males and females, and this is now mentioned in ‘Animals’ of the methods section (Line130).

The PSD labeling in Figure 5 is very difficult to resolve, even after increasing the magnification by 400%.

We apologize for the unclear images in the old Fig. 5. We have now enlarged the high magnification images and adjusted the intensity to clearly show the PSD.

Figure 6 - the addition of the data from the L4 cortical neurons does not add substantively to the paper since the timing of their development and the embryonic origin differs from that of the olfactory bulb neurons. Insight into the mechanisms involved is not clarified by the addition of the data from L4.

We believe that the data from L4 cortical neurons add substantively to the paper, as there have been many papers on synapse pruning during cortical development. These additional data strongly support the idea that microglia are generally dispensable for developmental (neurite-scale) dendrite pruning, contrary to a prevailing view. Mechanisms of dendrite pruning have been reported in a previous study (Fujimoto et al., Dev Cell 2023).

Reviewer #2

Mitral cells in the olfactory bulb and neurons in the barrel cortex undergo dendritic remodelling during development. One potential candidate to regulate this process is microglia, as it has been showed to be involved in synaptic pruning. Here, the authors convincingly demonstrate that microglia are not involved in dendritic and synaptic pruning in mitral cells and dendritic pruning in neurons in the barrel cortex. However, they found that microglia does regulate synaptic remodelling in granule cells in the olfactory bulb.

The findings are novel, interesting and useful for the field, and are based on robust data. The authors thoroughly explored every possible scenario in terms of pruning in mitral cells (dendritic, presynaptic, postsynaptic), delved into the olfactory circuitry (granule cells) and investigated the same process in another neuron type, in this case the neurons of the barrel cortex. For all the reasons above, I strongly recommend this article for publication.

We thank this reviewer for acknowledging our findings.

I have several minor comments:

- Please, replace any red channel in the figures by magenta, or another colour-blind friendly choice (like in Fig. 3).

We used magenta when combined with green (e.g., Fig. 3A, Fig. 4A, B, Fig. 5A). However, we would like to use red when combined with blue and without green, because magenta is difficult to see when merged with blue. Color-blind people would have no problem in this case.

- Fig. 1A, Supp. Fig. 1A, and Supp. Fig. 2A: please, specify ‘ICR’ abbreviation in figure legend.

It stands for ICR mice, but we have removed this unnecessary and confusing information from the figure.

- Supp. Fig. 1D, Supp. Fig. 2D: the authors should include the statistical tests in the quantifications. Were they not considered because they finally chose the most efficient inhibitor?

We agree. We have added the statistical tests. We have also updated these figure panels so that they appear consistent with the main Fig. 1D.

- Fig. 3: The authors show convincingly that dendritic pruning is unaffected between control and microglia-depleted conditions, both at P4 and P6. However, there are two percentages of neurons in the controls that do not end up projecting to a single glomerulus. Is this physiological/known? If so, could the authors indicate it briefly in the discussion/highlight the reference of the study?

Some mitral cells continue to innervate multiple glomeruli even at P28, as described in previous reports (Fujimoto et al., 2023). This is now explained in the main text (Line 269-270).

- Fig. 2, Fig. 3, Fig. 6: for the sake of consistency with previous figures, please indicate the number of mice used in these experiments.

We respectfully disagree. In these experiments, the sample sizes used for the statistical analyses are the number of “neurons”, not mice, because we care about variation among neurons. Therefore, it is best to report the number of neurons in figures. The number of animals is now given in the figure legends. We typically obtained samples (neurons) from several animals 3-6 mice.

- Fig. 3: for the sake of clarity, please add the ages in graphs of B and C (like the ones in Fig. 4).

Age is now added to the top of the panels.

- Fig. 6: like in Fig. 2, please add example images of electroporated neurons to accompany the schematics of the reconstructions.

We have now added raw fluorescence images associated with the reconstructed neurons.

- Methods, ‘Confocal imaging and image processing’ section: details about type of acquisitions (e.g., stacks) and pixel size should be added.

These details are added to the method section (Line 202-205).

We thank both reviewers for their constructive comments on this manuscript. We believe that both reviewers will be satisfied with this revision.

All the changes are now highlighted in the text.

## References

[B1] Aihara S, Fujimoto S, Sakaguchi R, Imai T (2021) BMPR-2 gates activity-dependent stabilization of primary dendrites during mitral cell remodeling. Cell Rep 35:109276. 10.1016/j.celrep.2021.109276 34161760

[B2] Antón-Bolaños N, Sempere-Ferràndez A, Guillamón-Vivancos T, Martini FJ, Pérez-Saiz L, Gezelius H, Filipchuk A, Valdeolmillos M, López-Bendito G (2019) Prenatal activity from thalamic neurons governs the emergence of functional cortical maps in mice. Science 364:987–990. 10.1126/science.aav7617 31048552 PMC7611000

[B3] Auguste YSS, Ferro A, Kahng JA, Xavier AM, Dixon JR, Vrudhula U, Nichitiu AS, Rosado D, Wee TL, Pedmale UV, Cheadle L (2022) Oligodendrocyte precursor cells engulf synapses during circuit remodeling in mice. Nat Neurosci 25:1735–1735. 10.1038/s41593-022-01209-z 36344700 PMC9708582

[B4] Awasaki T, Ito K (2004) Engulfing action of glial cells is required for programmed axon pruning during *Drosophila* metamorphosis. Curr Biol 14:668–677. 10.1016/j.cub.2004.04.001 15084281

[B5] Brown GC, Neher JJ (2014) Microglial phagocytosis of live neurons. Nat Rev Neurosci 15:209–216. 10.1038/nrn3710 24646669

[B6] Buchanan J, et al. (2022) Oligodendrocyte precursor cells ingest axons in the mouse neocortex. Proc Natl Acad Sci U S A 119:e2202580119. 10.1073/pnas.2202580119 36417438 PMC9889886

[B7] Buchanan J, da Costa NM, Cheadle L (2023) Emerging roles of oligodendrocyte precursor cells in neural circuit development and remodeling. Trends Neurosci 46:628–639. 10.1016/j.tins.2023.05.007 37286422 PMC10524797

[B8] Chen CF, Regehr WG (2000) Developmental remodeling of the retinogeniculate synapse. Neuron 28:955–966. 10.1016/s0896-6273(00)00166-5 11163279

[B9] Chung WS, Clarke LE, Wang GX, Stafford BK, Sher A, Chakraborty C, Joung J, Foo LC, Thompson A, Chen CF, Smith SJ, Barres BA (2013) Astrocytes mediate synapse elimination through MEGF10 and MERTK pathways. Nature 504:394–400. 10.1038/nature12776 24270812 PMC3969024

[B10] Dagher NN, Najafi AR, Kayala KMN, Elmore MRP, White TE, Medeiros R, West BL, Green KN (2015) Colony-stimulating factor 1 receptor inhibition prevents microglial plaque association and improves cognition in 3xTg-AD mice. J Neuroinflammation 12:139. 10.1186/s12974-015-0366-9 26232154 PMC4522109

[B11] Denizet M, Cotter L, Lledo PM, Lazarini F (2017) Sensory deprivation increases phagocytosis of adult-born neurons by activated microglia in the olfactory bulb. Brain Behav Immun 60:38–43. 10.1016/j.bbi.2016.09.015 27640898

[B12] Elmore MRP, Najafi AR, Koike MA, Dagher NN, Spangenberg EE, Rice RA, Kitazawa M, Matusow B, Nguyen H, West BL, Green KN (2014) Colony-stimulating factor 1 receptor signaling is necessary for microglia viability, unmasking a microglia progenitor cell in the adult brain. Neuron 82:380–397. 10.1016/j.neuron.2014.02.040 24742461 PMC4161285

[B13] Erzurumlu RS, Gaspar P (2012) Development and critical period plasticity of the barrel cortex. Eur J Neurosci 35:1540–1553. 10.1111/j.1460-9568.2012.08075.x 22607000 PMC3359866

[B14] Faust TE, Gunner G, Schafer DP (2021) Mechanisms governing activity-dependent synaptic pruning in the developing mammalian CNS. Nat Rev Neurosci 22:657–673. 10.1038/s41583-021-00507-y 34545240 PMC8541743

[B15] Figueres-Oñate M, López-Mascaraque L (2016) Adult olfactory bulb interneuron phenotypes identified by targeting embryonic and postnatal neural progenitors. Front Neurosci 10:194. 10.3389/fnins.2016.00194 27242400 PMC4860398

[B16] Fujimoto S, Leiwe MN, Aihara S, Sakaguchi R, Muroyama Y, Kobayakawa R, Kobayakawa K, Saito T, Imai T (2023) Activity-dependent local protection and lateral inhibition control synaptic competition in developing mitral cells in mice. Dev Cell 58:1221–1236.e7. 10.1016/j.devcel.2023.05.004 37290446

[B17] Ginhoux F, Greter M, Leboeuf M, Nandi S, See P, Gokhan S, Mehler MF, Conway SJ, Ng LG, Stanley ER, Samokhvalov IM, Merad M (2010) Fate mapping analysis reveals that adult microglia derive from primitive macrophages. Science 330:841–845. 10.1126/science.1194637 20966214 PMC3719181

[B18] Goodman CS, Shatz CJ (1993) Developmental mechanisms that generate precise patterns of neuronal connectivity. Cell 72 [Suppl]:77–98. 10.1016/s0092-8674(05)80030-3 8428376

[B19] Grier BD, Belluscio L, Cheetham CEJ (2016) Olfactory sensory activity modulates microglial-neuronal interactions during dopaminergic cell loss in the olfactory bulb. Front Cell Neurosci 10:178. 10.3389/fncel.2016.00178 27471450 PMC4945633

[B20] Guedes JR, Ferreira PA, Costa JM, Cardoso AL, Peça J (2022) Microglia-dependent remodeling of neuronal circuits. J Neurochem 163:74–93. 10.1111/jnc.15689 35950924 PMC9826178

[B21] Hayashi-Takagi A, Yagishita S, Nakamura M, Shirai F, Wu YI, Loshbaugh AL, Kuhlman B, Hahn KM, Kasai H (2015) Labelling and optical erasure of synaptic memory traces in the motor cortex. Nature 525:333–338. 10.1038/nature15257 26352471 PMC4634641

[B22] Imai T (2014) Construction of functional neuronal circuitry in the olfactory bulb. Semin Cell Dev Biol 35:180–188. 10.1016/j.semcdb.2014.07.012 25084319

[B23] Imai T, Yamazaki T, Kobayakawa R, Kobayakawa K, Abe T, Suzuki M, Sakano H (2009) Pre-target axon sorting establishes the neural map topography. Science 325:585–590. 10.1126/science.1173596 19589963

[B24] James KN, Throesch BT, Davini W, Eade KT, Ghosh S, Lee S, Torabi-Rander N, Baldwin KK (2017) Activity based checkpoints ensure circuit stability in the olfactory system. bioRxiv 156372.

[B25] Kanamori T, Kanai MI, Dairyo Y, Yasunaga K, Morikawa RK, Emoto K (2013) Compartmentalized calcium transients trigger dendrite pruning in *Drosophila* sensory neurons. Science 340:1475–1478. 10.1126/science.1234879 23722427

[B26] Kanamori T, Yoshino J, Yasunaga K, Dairyo Y, Emoto K (2015) Local endocytosis triggers dendritic thinning and pruning in *Drosophila* sensory neurons. Nat Commun 6:6515. 10.1038/ncomms7515 25761586

[B27] Katz LC, Shatz CJ (1996) Synaptic activity and the construction of cortical circuits. Science 274:1133–1138. 10.1126/science.274.5290.1133 8895456

[B28] Ke MT, Imai T (2018) Optical clearing and index matching of tissue samples for high-resolution fluorescence imaging using SeeDB2. Bio Protoc 8:e3046. 10.21769/BioProtoc.3046 34532520 PMC8342116

[B29] Ke MT, Nakai Y, Fujimoto S, Takayama R, Yoshida S, Kitajima TS, Sato M, Imai T (2016) Super-resolution mapping of neuronal circuitry with an index-optimized clearing agent. Cell Rep 14:2718–2732. 10.1016/j.celrep.2016.02.057 26972009

[B30] Kelsch W, Lin CW, Mosley CP, Lois C (2009) A critical period for activity-dependent synaptic development during olfactory bulb adult neurogenesis. J Neurosci 29:11852–11858. 10.1523/JNEUROSCI.2406-09.2009 19776271 PMC2773669

[B31] Kettenmann H, Hanisch UK, Noda M, Verkhratsky A (2011) Physiology of microglia. Physiol Rev 91:461–553. 10.1152/physrev.00011.2010 21527731

[B32] Kurematsu C, et al. (2022) Synaptic pruning of murine adult-born neurons by microglia depends on phosphatidylserine. J Exp Med 219:e20202304. 10.1084/jem.20202304 35297954 PMC9195048

[B33] Lichtman JW, Colman H (2000) Synapse elimination and indelible memory. Neuron 25:269–278. 10.1016/s0896-6273(00)80893-4 10719884

[B34] Lin DM, Wang F, Lowe G, Gold GH, Axel R, Ngai J, Brunet L (2000) Formation of precise connections in the olfactory bulb occurs in the absence of odorant-evoked neuronal activity. Neuron 26:69–80. 10.1016/s0896-6273(00)81139-3 10798393

[B35] Luo LQ, O’Leary DDM (2005) Axon retraction and degeneration in development and disease. Annu Rev Neurosci 28:127–156. 10.1146/annurev.neuro.28.061604.135632 16022592

[B36] Malun D, Brunjes PC (1996) Development of olfactory glomeruli: temporal and spatial interactions between olfactory receptor axons and mitral cells in opossums and rats. J Comp Neurol 368:1–16. 10.1002/(SICI)1096-9861(19960422)368:1<1::AID-CNE1>3.0.CO;2-7 8725290

[B37] Matsui A, Tran M, Yoshida AC, Kikuchi SS, Mami U, Ogawa M, Shimogori T (2013) BTBD3 controls dendrite orientation toward active axons in mammalian neocortex. Science 342:1114–1118. 10.1126/science.1244505 24179155

[B38] Miyamoto A, Wake H, Ishikawa AW, Eto K, Shibata K, Murakoshi H, Koizumi S, Moorhouse AJ, Yoshimura Y, Nabekura J (2016) Microglia contact induces synapse formation in developing somatosensory cortex. Nat Commun 7:12540. 10.1038/ncomms12540 27558646 PMC5007295

[B39] Mizuno H, Luo W, Tarusawa E, Saito YM, Sato T, Yoshimura Y, Itohara S, Iwasato T (2014) NMDAR-regulated dynamics of layer 4 neuronal dendrites during thalamocortical reorganization in neonates. Neuron 82:365–379. 10.1016/j.neuron.2014.02.026 24685175

[B40] Mizuno H, Ikezoe K, Nakazawa S, Sato T, Kitamura K, Iwasato T (2018) Patchwork-type spontaneous activity in neonatal barrel cortex layer 4 transmitted via thalamocortical projections. Cell Rep 22:123–135. 10.1016/j.celrep.2017.12.012 29298415

[B41] Murai A, Iwata R, Fujimoto S, Aihara S, Tsuboi A, Muroyama Y, Saito T, Nishizaki K, Imai T (2016) Distorted coarse axon targeting and reduced dendrite connectivity underlie dysosmia after olfactory axon injury. eNeuro 3:ENEURO.0242-16.2016. 10.1523/ENEURO.0242-16.2016 PMC5066264 27785463

[B42] Nagappan-Chettiar S, Yasuda M, Johnson-Venkatesh EM, Umemori H (2023) The molecular signals that regulate activity-dependent synapse refinement in the brain. Curr Opin Neurobiol 79:102692. 10.1016/j.conb.2023.102692 36805716 PMC10023433

[B43] Nakazawa S, Iwasato T (2021) Spatial organization and transitions of spontaneous neuronal activities in the developing sensory cortex. Dev Growth Differ 63:323–339. 10.1111/dgd.12739 34166527

[B44] Paolicelli RC, Bolasco G, Pagani F, Maggi L, Scianni M, Panzanelli P, Giustetto M, Ferreira TA, Guiducci E, Dumas L, Ragozzino D, Gross CT (2011) Synaptic pruning by microglia is necessary for normal brain development. Science 333:1456–1458. 10.1126/science.1202529 21778362

[B45] Rappert A, Bechmann I, Pivneva T, Mahlo J, Biber K, Nolte C, Kovac AD, Gerard C, Boddeke HWGM, Nitsch R, Kettenmann H (2004) CXCR3-dependent microglial recruitment is essential for dendrite loss after brain lesion. J Neurosci 24:8500–8509. 10.1523/JNEUROSCI.2451-04.2004 15456824 PMC6729901

[B46] Riccomagno MM, Kolodkin AL (2015) Sculpting neural circuits by axon and dendrite pruning. Annu Rev Cell Dev Biol 31:779–805. 10.1146/annurev-cellbio-100913-013038 26436703 PMC4668927

[B47] Rosin JM, Vora SR, Kurrasch DM (2018) Depletion of embryonic microglia using the CSF1R inhibitor PLX5622 has adverse sex-specific effects on mice, including accelerated weight gain, hyperactivity and anxiolytic-like behaviour. Brain Behav Immun 73:682–697. 10.1016/j.bbi.2018.07.023 30056204

[B48] Sapar ML, Ji H, Wang B, Poe AR, Dubey K, Ren XJ, Ni JQ, Han C (2018) Phosphatidylserine externalization results from and causes neurite degeneration in. Cell Rep 24:2273–2286. 10.1016/j.celrep.2018.07.095 30157423 PMC6174084

[B49] Schafer DP, Lehrman EK, Kautzman AG, Koyama R, Mardinly AR, Yamasaki R, Ransohoff RM, Greenberg ME, Barres BA, Stevens B (2012) Microglia sculpt postnatal neural circuits in an activity and complement-dependent manner. Neuron 74:691–705. 10.1016/j.neuron.2012.03.026 22632727 PMC3528177

[B50] Scott-Hewitt N, Perrucci F, Morini R, Erreni M, Mahoney M, Witkowska A, Carey A, Faggiani E, Schuetz LT, Mason S, Tamborini M, Bizzotto M, Passoni L, Filipello F, Jahn R, Stevens B, Matteoli M (2020) Local externalization of phosphatidylserine mediates developmental synaptic pruning by microglia. EMBO J 39:e105380. 10.15252/embj.2020105380 32657463 PMC7429741

[B51] Stanley ER, Chitu V (2014) CSF-1 receptor signaling in myeloid cells. Cold Spring Harb Perspect Biol 6:a021857. 10.1101/cshperspect.a021857 24890514 PMC4031967

[B52] Stevens B, Allen NJ, Vazquez LE, Howell GR, Christopherson KS, Nouri N, Micheva KD, Mehalow AK, Huberman AD, Stafford B, Sher A, Litke AM, Lambris JD, Smith SJ, John SWM, Barres BA (2007) The classical complement cascade mediates CNS synapse elimination. Cell 131:1164–1178. 10.1016/j.cell.2007.10.036 18083105

[B53] St John JA, Key B (2002) Heterogeneity in olfactory neurons in mouse revealed by differential expression of glycoconjugates. Histochem J 34:281–289. 10.1023/a:1023374407724 12769259

[B54] Tasdemir-Yilmaz OE, Freeman MR (2014) Astrocytes engage unique molecular programs to engulf pruned neuronal debris from distinct subsets of neurons. Genes Dev 28:20–33. 10.1101/gad.229518.113 24361692 PMC3894410

[B55] Thion MS, Ginhoux F, Garel S (2018) Microglia and early brain development: an intimate journey. Science 362:185–189. 10.1126/science.aat0474 30309946

[B56] Vanderhaeghen P, Cheng HJ (2010) Guidance molecules in axon pruning and cell death. Cold Spring Harb Perspect Biol 2:a001859. 10.1101/cshperspect.a001859 20516131 PMC2869516

[B57] Wallace J, Lord J, Dissing-Olesen L, Stevens B, Murthy VN (2020) Microglial depletion disrupts normal functional development of adult-born neurons in the olfactory bulb. Elife 9:e50531. 10.7554/eLife.50531 32150529 PMC7062469

[B58] Watanabe M, Kano M (2011) Climbing fiber synapse elimination in cerebellar Purkinje cells. Eur J Neurosci 34:1697–1710. 10.1111/j.1460-9568.2011.07894.x 22103426

[B59] Wong ROL, Ghosh A (2002) Activity-dependent regulation of dendritic growth and patterning. Nat Rev Neurosci 3:803–812. 10.1038/nrn941 12360324

