# Supplementary figures and images for: Microglia Are Dispensable for Developmental Dendrite Pruning of Mitral Cells in Mice
Source: eNeuro. 2023 Nov 8;10(11):ENEURO.0323-23.2023. doi: 10.1523/ENEURO.0323-23.2023 (PMC10644373; doi:10.1523/ENEURO.0323-23.2023)

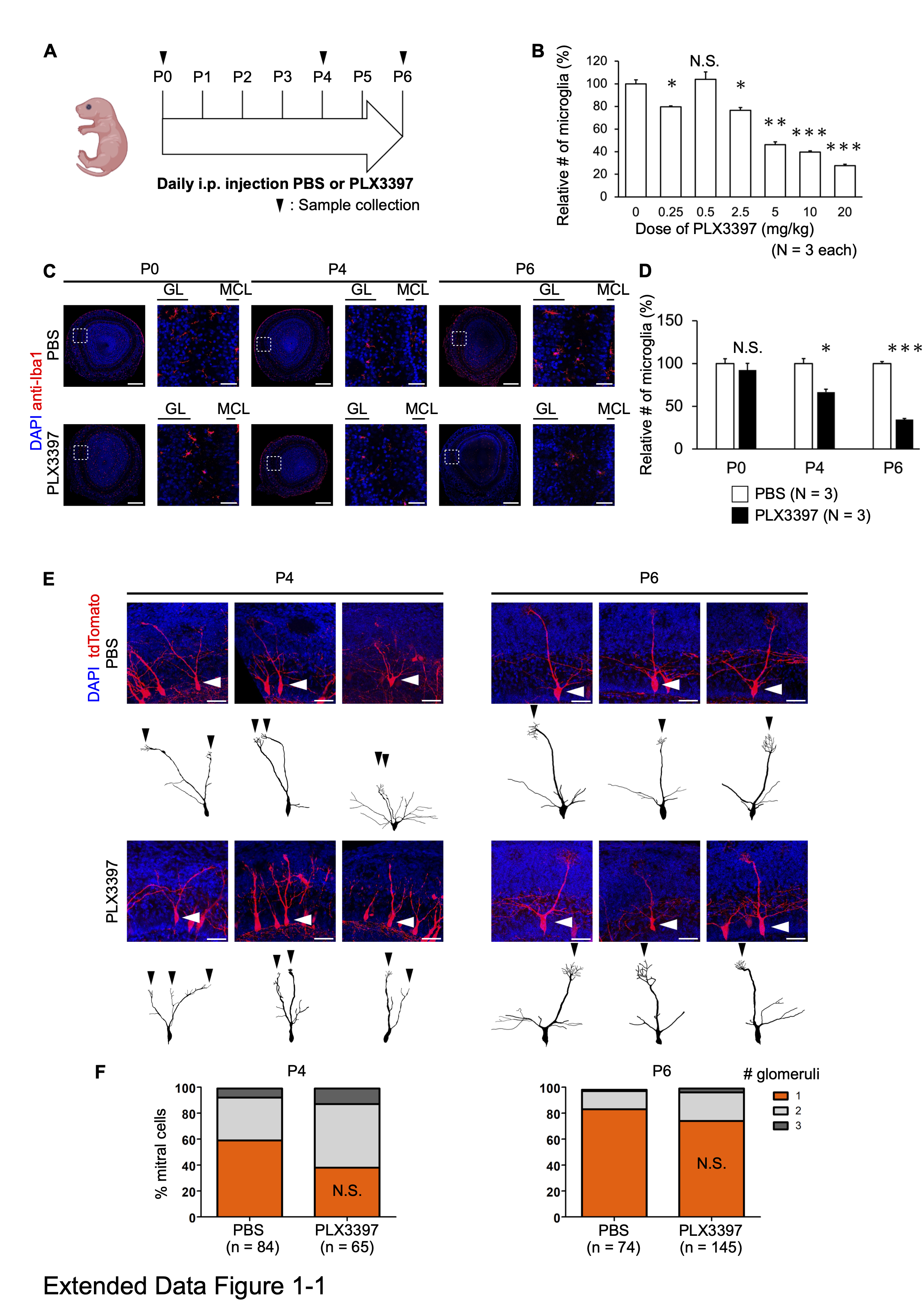

Supplement: Extended Data Figure 1-1 — Microglial depletion by postnatal PLX3397 treatment. A, Timeline of PLX3397 treatment in postnatal mice. PLX3397 was intraperitoneally injected to pups twice a day from P0 to P6. Mice were analyzed at P0, P4, and P6. B, Relative number of microglia at P6 after treatment with different doses of PLX3397. The x-axis shows the dose of PLX3397 injection (mg/kg body weight). ***p < 0.001, **p < 0.01, *p < 0.05 (Welch’s t test, compared with the control). C, Anti-Iba1 staining in the olfactory bulb of control and PLX3397-treated (20 mg/kg body weight) mice. Scale bars represent 300 μm (left) and 50 μm (right). D, Relative number of microglia at different stages. ***p < 0.001, *p < 0.05 (Welch’s t test, compared with the control). E, Representative traces of mitral cells in control and PLX3397-treated mice. F, Quantification of glomeruli innervated by individual mitral cells. N.S., nonsignificant (χ2 test compared to the control). Number of neurons (n) are indicated in parentheses. Data are from three or four mice per group. Download Figure 1-1, TIF file. [file enu-eN-NWR-0323-23-s01.tif]
